# Applications and User Perceptions of Smart Glasses in Emergency Medical Services: Semistructured Interview Study

**DOI:** 10.2196/30883

**Published:** 2022-02-28

**Authors:** Zhan Zhang, Karen Joy, Richard Harris, Mustafa Ozkaynak, Kathleen Adelgais, Kevin Munjal

**Affiliations:** 1 School of Computer Science and Information Systems Pace University New York, NY United States; 2 College of Nursing University of Colorado Aurora, CO United States; 3 School of Medicine University of Colorado Aurora, CO United States; 4 Department of Emergency Medicine Mount Sinai Medical Center New York, NY United States

**Keywords:** smart glasses, hands-free technologies, emergency medical services, user studies, mobile phone

## Abstract

**Background:**

Smart glasses have been gaining momentum as a novel technology because of their advantages in enabling *hands-free* operation and *see-what-I-see* remote consultation. Researchers have primarily evaluated this technology in hospital settings; however, limited research has investigated its application in prehospital operations.

**Objective:**

The aim of this study is to understand the potential of smart glasses to support the work practices of prehospital providers, such as emergency medical services (EMS) personnel.

**Methods:**

We conducted semistructured interviews with 13 EMS providers recruited from 4 hospital-based EMS agencies in an urban area in the east coast region of the United States. The interview questions covered EMS workflow, challenges encountered, technology needs, and users’ perceptions of smart glasses in supporting daily EMS work. During the interviews, we demonstrated a system prototype to elicit more accurate and comprehensive insights regarding smart glasses. Interviews were transcribed verbatim and analyzed using the open coding technique.

**Results:**

We identified four potential application areas for smart glasses in EMS: enhancing teleconsultation between distributed prehospital and hospital providers, semiautomating patient data collection and documentation in real time, supporting decision-making and situation awareness, and augmenting quality assurance and training. Compared with the built-in touch pad, voice commands and hand gestures were indicated as the most preferred and suitable interaction mechanisms. EMS providers expressed positive attitudes toward using smart glasses during prehospital encounters. However, several potential barriers and user concerns need to be considered and addressed before implementing and deploying smart glasses in EMS practice. They are related to hardware limitations, human factors, reliability, workflow, interoperability, and privacy.

**Conclusions:**

Smart glasses can be a suitable technological means for supporting EMS work. We conclude this paper by discussing several design considerations for realizing the full potential of this hands-free technology.

## Introduction

### Background

Prehospital care is a high-risk, time-sensitive medical domain where first responders such as emergency medical services (EMS) providers provide urgent care to patients in the field and transport them to the nearest hospital or care facility. The primary goal of prehospital care is to stabilize patients by quickly addressing severe illnesses or life-threatening injuries. Perhaps prehospital care is among the most challenging medical domains in the provision of care to patients owing to various reasons, such as the broad range of clinical situations, limited resources and time, difficulties in accessing remote experts, and the highly dynamic situations and environmental conditions that providers encounter [1,2]. Owing to such challenges, technology support could be useful for EMS providers to facilitate decision-making and information management [3,4]. Despite some efforts, the prehospital environment remains one of the few medical settings with limited technology support [2]. In addition, previous work has primarily focused on developing and implementing systems on conventional handheld devices such as tablets or smartphones. For example, in an early study, Tollefsen et al [5] developed a menu-driven mobile app for EMS teams to document patient information, which was then uploaded to a central database for hospital care providers to instantaneously access and review. Another study designed and evaluated a smartphone app to facilitate care documentation in the field by enabling EMS providers to photograph the patient, record digital audio notes, and capture the view of the incident [6]. Despite their beneficial features, these handheld devices could cause problems in real-time use because (1) handheld devices are prone to interfere with manual tasks in a busy EMS environment [7-11] and (2) the physical handling of these devices could increase the chance of cross-contamination and patient infections [12]. As such, the need for novel technologies to support hands-busy EMS operations is evident [2,13].

Since being introduced to the public in 2011, smart glasses (a wearable technology in the shape of conventional glasses with a transparent screen and a video camera) have been gaining momentum because they can offer hands-free operation through novel interaction mechanisms such as voice control [14-18]. Google Glass has received the most exposure initially and stimulated the development of smart glasses by the industry. Compared with handheld devices, smart glasses enable constant information presentation and access in a hands-free manner and allow local workers to project first-person point-of-view to a remote viewer. Given these benefits, researchers have been exploring their potential in clinical and surgical environments [16], such as surgical telementoring [19-21], remote evaluation of patients with acute medical conditions [22], and disaster triage [23,24]. This body of literature demonstrated that smart glasses are potentially useful in supporting care management [16,17,25-28] and can enable secure Health Insurance Portability and Accountability Act (HIPAA)-compliant communications [29].

### Research Gaps and Study Objective

Smart glasses can particularly benefit EMS as hands-free interaction can be a useful resource to handle situations with a lot of uncertainty. However, research on smart glasses in the prehospital environment is limited, with a few notable exceptions [13,23,24,30,31]. In addition, the previous work primarily focused on developing smart glass apps for certain EMS scenarios, such as disaster telemedicine triage [23,24], patient localization [30], and mobile vital sign monitoring [13]. To gain a comprehensive understanding of the application areas of smart glasses in EMS, the barriers and user concerns related to the use of smart glasses in practice, and how best to integrate this novel technology into the current EMS workflow, we conducted interviews to explore the potential and affordances of smart glasses in the out-of-hospital setting from the perspective of EMS providers to derive design implications for this novel technology. This study is part of a larger research effort that aims to iteratively design, develop, and evaluate smart glass technologies to support EMS operations in the field. In this work, we aim to answer the following three research questions through interviews with EMS providers: (1) How can smart glasses support EMS providers in overcoming challenges in the prehospital setting? (2) What interaction modality (eg, voice control, touch, and hand gestures) is most preferred and appropriate? (3) What types of concerns or potential barriers could impede the adoption and real-time use of this novel technology by EMS providers?

## Methods

### Study Design

We used a qualitative study approach (eg, interviews) [32,33] to gain an empirical and in-depth understanding of EMS providers’ perceptions of and needs for adopting smart glasses in their daily work. This study approach has been successful in informing the design of complex sociotechnical systems [34]. The interview guide (Multimedia Appendix 1) was informed by previous work [35] and was developed in an iterative manner by the researchers. We also pilot-tested the interview guide with 2 experts (ie, EMS team leaders) to ensure the clarity, appropriateness, and relevance of the questions.

### Participants

We conducted semistructured interviews with 13 EMS providers recruited from 4 hospital-based EMS agencies in an urban area in the east coast region of the United States. As shown in Table 1, a total of 85% (11/13) of them are paramedics, whereas the remaining 15% (2/13) are emergency medical technicians. Their years of experience ranged from 4 to 30 years, with 15% (2/13) of the participants being EMS directors. In addition, a few of them also serve other roles, such as EMS operation manager and quality assurance coordinator.

**Table 1:** Participant demographics (N=13).

ID	Sex	Occupation	Years of experience
P1	Male	Paramedic	28
P2	Male	Paramedic and EMS^a^ educator	15
P3	Male	Paramedic and EMS director	25
P4	Male	Paramedic	18
P5	Male	Paramedic and quality assurance coordinator	30
P6	Male	Paramedic and EMS director	>30
P7	Female	Emergency medical technician	11
P8	Male	Paramedic	23
P9	Male	Paramedic	14
P10	Male	Emergency medical technician	4
P11	Male	Paramedic and EMS operation manager	21
P12	Male	Paramedic	11
P13	Male	Paramedic	7

^a^EMS: emergency medical services.

We included both emergency medical technicians and paramedics in our study because they represent the major types of EMS providers in the United States. Emergency medical technicians are trained to provide basic life support such as oxygen administration, wound treatment, and cardiopulmonary resuscitation. In contrast, the scope of practice and autonomy of paramedics are greater. Paramedics are allied health professionals with >1000 hours of training and provide advanced life support for patients, including advanced airway management, electrocardiogram interpretation, and medication administration. With both emergency medical technician and paramedic roles involved in our study, we were able to gain a holistic understanding of the use scenarios of smart glasses from different perspectives.

### Data Collection

Owing to the COVID-19 pandemic, we conducted interviews via Zoom (Zoom Video Communications) following the best practices and experiences shared by other researchers who had to transition their user studies from in-person settings to web-based environments [36]. The interviews were conducted by two trained researchers (ZZ and KJ) and lasted for 45 to 90 minutes. Each interview was roughly divided into three sections: the first section consisted of general questions related to participant’s demographics, work experience, and education or training background; the second section focused on EMS workflow from dispatch to patient hand off, artifacts and digital tools used in practice, and challenges encountered in their work (eg, documentation, care coordination, and communication with patients, dispatchers, and remote experts); the third section inquired about EMS professional’s perceptions of using smart glasses in their daily work.

**Figure 1:**
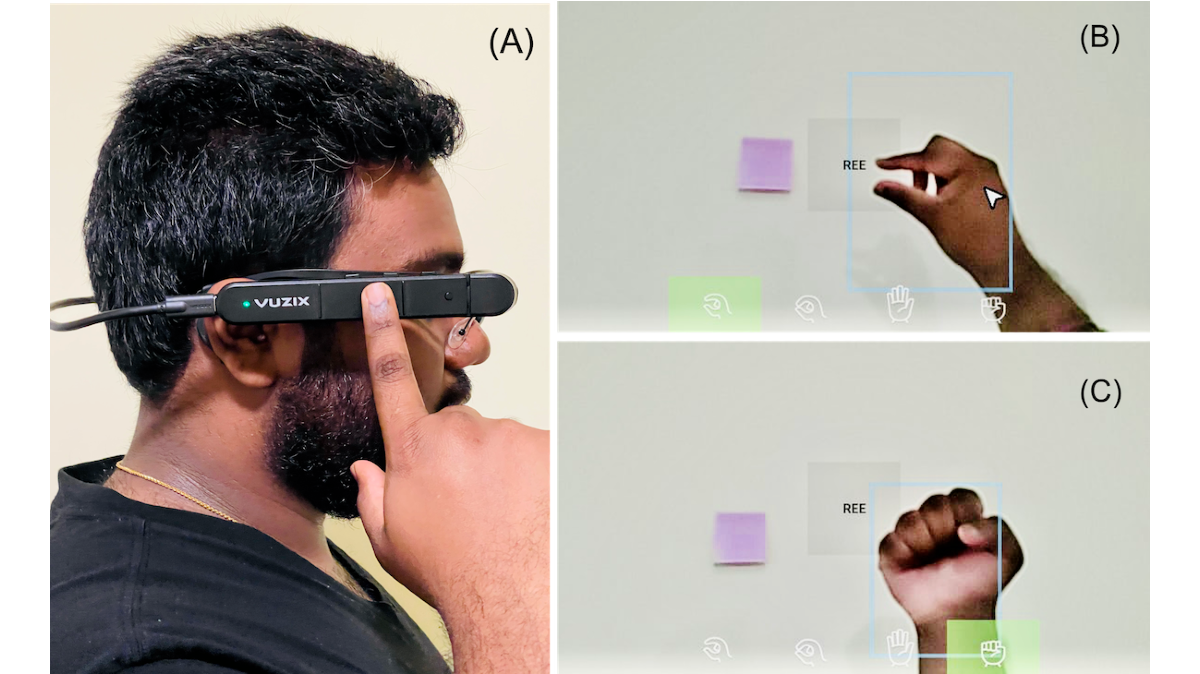
Interaction modalities: (A) Use the built-in touch pad to navigate the user interface of smart glasses. (B) Perform an open pinch to select the cursor on the glass screen. (C) Present an open hand and close it to navigate back to the home screen.

To help participants better understand this novel technology (eg, how it looks like and how it works) and elicit accurate and comprehensive insights, we used the Vuzix M400 [37] product to explain the hardware and software components of the device and possible interaction modalities through video and live demonstrations. In particular, we illustrated 3 modalities to interact with smart glasses. The first interaction modality was via the built-in touch pad and navigation buttons, which require physical touching and clicking on the device (Figure 1A). The second modality was voice commands created through the Vuzix software development kit. The third modality we demonstrated was hand gestures–based interaction, such as performing an open pinch to select the cursor (Figure 1B) and presenting an open hand and closing it to navigate back to the home page (Figure 1C). We implemented this interaction mechanism using the software development kit provided by CrunchFish [38], a Swedish technology company that develops gesture recognition software for mobile and wearable devices. We concluded the system demonstration by briefly discussing about its current application in other medical domains, such as in the operating room, wound care, and disaster triage.

### Data Analysis

All interviews were audio-recorded and transcribed verbatim for analysis. Anonymized interview transcripts were analyzed by two researchers (ZZ and KJ) using an open coding technique [39]. We chose this coding technique because it could generate rich and detailed insights of participants’ perspectives and opinions through iterative-inductive analysis of interview transcripts [40]. More specifically, the researchers first reviewed an initial set of transcripts independently (3/13, 23%). Then, the initial list of codes was generated and discussed among researchers to determine which codes to retain, merge, or remove. After the list of codes was set, we created a codebook to define each code to standardize the following coding process. The codebook was developed in an iterative manner until a consensus was reached. Then, the 2 researchers analyzed the remaining transcripts using the codebook. They met regularly to discuss and compare their codes for each interview transcript. The disagreements were resolved through discussion. Then, following a thematic analysis approach [41,42], the codes were grouped into themes using affinity diagrams [43], a common approach for creating connections or finding patterns in qualitative data. This step allowed the researchers to identify overarching themes describing EMS providers’ opinions about the application areas of smart glasses in EMS operation, preferred interaction modalities, and potential barriers to adopting this novel technology. We have discussed about these major themes in the following section.

### Ethical Consideration

This study was approved by the Pace University institutional review board (1515261). All participants provided their consent to participate in the study and be audio-recorded.

## Results

### Application Areas of Smart Glasses in EMS Work

#### Overview

The EMS participants identified a set of potential application areas where smart glasses could facilitate their work. We categorized them into four areas: teleconsultation, documentation, decision support, and quality assurance and training. We describe each use scenario in detail in the following sections.

#### Teleconsultation

The most prominent application area of smart glasses raised by EMS providers was teleconsultation. The reason is that EMS providers sometimes need to talk to a remote expert (eg, emergency department [ED] physician) for consultation, such as getting permission or advice for medication administration and collectively understanding the patient’s status to decide the next steps. Currently, they rely solely on traditional communication mechanisms, such as radio or phone, to share and discuss information. However, these mechanisms have their intrinsic limitations, posing challenges for efficient communication between distributed EMS and ED teams. That is, in the field, EMS providers need to describe with words the situation they face; on the other side, ED physicians at the hospital often have difficulties in understanding what is precisely happening in the field potentially owing to ambient noise or disruptions in connectivity. As 1 participant explained:

Physicians maybe not hearing details correctly because it is over the radio. There’s always going to be lag or miscommunication when you are using radios to relay information.

P8

As such, our participants expressed the emerging need for visual-based technologies to support their communication with the hospital. They believed that smart glasses could serve as an unobtrusive technological means to improve the communication and care coordination between prehospital and hospital care providers, as 1 participant explained:

I think it would be a useful tool, especially in those situations where you are going to end up contacting a physician, and they can actually see the environment in which the patient is. They can see the patient, specifically stroke patients, so that the physician can actually see the patient and facial droop and actually look at the patient. So, for those situations, I think it would be very helpful...You can have a conversation with the physician. Might be helpful for the physician to see the patient and what is being done to the patient at the same time for their purpose of understanding and getting a better picture of what’s going on. I think it will be very helpful.

P1

#### Documentation

During the interviews, several participants identified the potential use of smart glasses in documentation. For example, participants stated that they could use smart glasses to take pictures and videos, which can be saved in the electronic health record (EHR) system to document patient injuries and wounds. In addition, these context-rich data can be shared with ED physicians to help them understand the severity of patient injury:

If you were able to do like a real-time video recording for a trauma patient to document like the mechanism of injury, like for falls or for car accident, and to be able to show those to the clinicians at the hospital, the doctors would love that.

P4

Another specific use for documentation is allowing EMS providers to dictate to the smart glasses and have the smart glasses transcribe the dictation to text through voice recognition. This use case was seen as a potential facilitator of documentation in the field, which could save a significant amount of time and efforts that can be spent on patient care:

I think it will be helpful for actual data collection for timing and all that. I think it’s an excellent tool because it will make life easier if they [EMS providers] could actually just dictate certain things and they can be automatically stored in the electronic medical record.

P5

The ability to scan medications was also deemed useful by several participants. This feature could allow EMS providers to scan the barcode of the medication given to the patient, and the detailed information of the medication (eg, name and dosage) is automatically saved to EHR. In addition, as some patients could take several medications for chronic disease management and EMS providers may not have sufficient time to capture all the details, the medication scanning feature enabled by smart glasses could also make the collection of patient’s medical history much easier:

Sometimes people come to us with a bag of medication and they're like, these are my medications. So, if I could just turn on the glasses and scan them and I have all the medications there...I mean, it'll not only make our jobs a little easier, but also expedite our patient’s transport to the hospital.

P7

#### Decision Support

Participants believed that smart glasses could become a powerful decision support tool. For example, embedding medical protocols in the app could help EMS providers to perform a range of complex medical activities, verify the steps of less frequent tasks, and ensure compliance with medical procedures. One participant explained:

I think it would be great to integrate a point of reference to it (smart glass). Like, you know, we have protocols. Sometimes not everybody is going to have the same types of calls. Some people rarely get aphylactic calls, so when they get it, they would be like “oh my god, how much do I give?”...If you are newer in the field or you don’t know your protocol because you’ve never encountered this problem, it would be great just to pull up that reference.

P7

Other mentioned decision support opportunities included augmenting information searching in a hands-free manner (P10), facilitating the determination of medication or fluid dosage (P12), and constantly presenting vital signs information to enhance situation awareness (P13):

Have a way to find references to normal vital signs or to look up definitions or features of different medical conditions.

P10

I think where the smart glasses could be very, very helpful is, as like a second check before you administer narcotics...If I don't have to take out my cell phone to confirm weight, pounds to kilograms and like, do the conversions, if I could just do that to my smart glasses without touching anything, I think that would be like perfect.

P12

If you were working on a cardiac arrest and the monitors not facing you, you can use the smart glass to pull up the vital signs using this head-up display. It would be awesome.

P13

#### Quality Assurance and Training

Participants considered smart glasses as a useful tool to record either the entire patient care process or critical medical procedures (eg, how the patient was treated for a complication in the field). This use of smart glasses can potentially enhance the quality of care by urging EMS providers to be more compliant:

If things are being recorded, it might make sure that you follow a protocol very correctly. With a digital record of when and how you do things, you’re a lot more likely to kind of do things closer to the textbook.

P13

Furthermore, the video recordings of patient care can be used for both quality assurance and training purposes:

It’ll be very good for educational quality assurance. You can actually trim videos for different things that went wrong, and you could use it to instruct your staff.

P5

Another interesting application area mentioned by a participant was helping with litigation issues faced by EMS providers in their work:

A lot of times, if something happens, you can be accused. But if you can have a recording with the voice, it could be used to protect the crews from litigation.

P5

### Preferred Interaction Modalities

Participants were asked to rank the 3 demonstrated interaction modalities from most preferred to least preferred. Our data show that 46% (6/13) of the participants ranked voice commands and hand gestures equally as the most favored interaction mechanism:

I’d probably be an even mix of hand gestures and voice commands if that was like being used practically in real life. I think the voice control would be the preferred way to control the device, but in a loud situation the voice can probably be a little bit clunky. It’d be easier to use hand gestures to accomplish the same thing.

P13

Among the rest of the participants (7/13, 54%), 57% (4/7) of them chose voice commands as the most preferred modality, whereas 29% (2/7) of them voted the hand gesture modality.

In contrast, touch pad was indicated as the least preferred interaction modality, with only 14% (1/7) of the participants choosing this mechanism over the others. The reason for not favoring touch pad was either because it occupied hands or owing to concerns about cross-contamination:

Touch I think it's definitely bad cause your hands are always disgusting. Plus, with COVID going on right now, you touch one surface, then you're touching something that's very close to your face [smart glasses], that is not safe.

P11

Despite the various views on these interaction modalities, a few participants highlighted the importance of having all the interaction modalities available so that they can choose which one to use depending on the situation:

You have to have redundancy, you have to have a backup. So, let's say, it's not responding to your voice, then you can do the touch or whatever.

P6

### Perceptions, Needs, and Concerns of Using Smart Glasses in Practice

#### Overview

Overall, our participants had a very positive attitude toward the use of smart glasses during prehospital encounters. Almost all of them expressed the willingness to use this system given its potential benefits; for example, not only supporting their work but also enhancing patient-centered care:

That would save so much time for the patient...So, you can do anything you got to do with your hands and it [smart glasses] will expedite the patient’s care, which leads to a better patient outcome. I would definitely use it.

P7

Despite the positive attitude, participants shared several concerns about deploying this novel technology in practice. We grouped the major concerns into six categories, including hardware limitations, human factors, reliability, workflow, interoperability, and privacy.

#### Hardware Limitations

Battery life of smart glasses was a major concern:

They need to be able to last a while, making sure that it's not like going to run out on me in the middle of a high acuity situation.

P12

Participants suggested installing a charging station inside the ambulance and preparing 1-2 backup batteries to ensure that the smart glasses can constantly run through a whole work shift; that is, 8 hours.

Durability was another common concern expressed by participants because the prehospital environment is messy and fast-paced, where any device can easily get lost or broken:

The first impression to me is if it could survive in the 911 system...I constantly have equipment that breaks and malfunctions. It is unfortunately that the city 911 system is very rugged, abusive and tough. So, you need to make sure that something is tough and durable then you might stand the chance.

P3

Finally, smart glasses require a high-bandwidth cellular network to establish video calls; however, some areas (eg, rural areas and subway stations) rarely have high-speed network access. As such, use of smart glasses for teleconsultation could be impacted sometimes. Similarly, participants were concerned about not being able to transfer the recorded data from smart glasses to other devices (eg, EHR system) in a timely manner—an issue that is often caused by connection failure between devices. Therefore, a few participants mentioned that smart glasses should have sufficient internal memory to allow *store-and-forward*, a common data transferring method in many telemedicine systems [44]:

If I’m in subway and there is no WIFI, and if I record videos or take pictures, or you know, telling it to dictate something, it should have enough internal memory to store everything recorded.

P5

Other hardware-related factors that might impact the adoption of smart glasses included device cost (eg, “my only concern is how much will this cost per unit?” [P1]), process of disinfecting the device (eg, “If it gets contaminated, how do you clean it?” [P5]), and safety issues (eg, “I’m definitely concerned about getting assaulted with it on.” [P9]).

#### Human Factors

Our participants raised several issues with regard to human factors. For example, 15% (2/13) of the participants commented that smart glasses could block a certain field of vision and, in turn, affect their patient care activities:

I think it could impede work if it's obstructing my view. I think that the most likely scenario where there would truly be an impedance to patient care is intubating a patient because I need to make sure that I have good vision of the patient’s vocal cords.

P12

In addition, it is vital to ensure that the device is not intrusive because “after a certain amount of time, stuff on your head could get irritating or annoying” [P5].

Compatibility with users’ own glasses or personal protective equipment was also frequently mentioned by our participants:

I like to wear goggles now, especially because of Covid-19. You need to make sure that the glasses have a good fit with the rest of your PPE, whether it’s a goggle or a face mask.

P12

#### Reliability

The smart glass app must be reliable because EMS work is high-acuity and time-critical; any system malfunction could lead to increased stress and high cognitive workload on EMS providers and even adverse patient outcomes. As such, system reliability is one of the primary concerns expressed by multiple participants:

If there is a technological failure, what is our backup, what do we do?

P4

#### Workflow

As our participants had little experience with smart glasses (compared with mobile phones or tablets), they were not clear whether this novel technology can seamlessly fit into EMS workflow:

Just like with every technology, just making sure that it is seamless and actually works. All of our technologies make sense in theory, but the application can be a little bit difficult.

P12

Another participant shared the same opinion and further commented that smart glasses might add more workload, such as the need to check the accuracy of recorded data when using it for documentation:

Obviously they [EMS providers] have to make sure that the information was recorded correctly. Is that another component that’s going to add time?

P3

#### Interoperability

Interoperability was also mentioned by the participants. For example, smart glasses should be integrated with the EHR system to realize the documentation use. Similarly, timely and constant data exchange (eg, electrocardiography, blood pressure, and oxygen saturation) between smart glasses and the vital signs monitor is critical for implementing the decision support feature. As such, the interoperability between smart glasses and other medical devices is essential:

I guess compatibility to different devices is very important. You know, trying to integrate something as simple as a monitor to any sort of technology is a bit of a hurdle because they don’t play nicely with each other.

P2

#### Privacy

Not surprisingly, data privacy was one of the most prominent concerns shared by almost all participants. Given that smart glasses would transfer, retrieve, and even store sensitive patient data, it is imperative to ensure compliance with the HIPAA regulations:

The number one concern would be patient privacy. That would have to be worked out. How is the data getting stored? How is the data getting processed?

P4

They also have concerns that patients, especially pediatric patients, might feel uncomfortable or nervous while seeing them wear a pair of smart glasses, as this device is rarely seen in daily life. Sometimes, patients may not even want to be recorded, so participants suggested that new regulations or rules regarding the digital recording of patients should be established before deploying the system in the field:

You always might have some patients who may not want to be recorded unless that’s the policy...you may also need to get patient’s consent and it becomes the legal issue.

P1

Finally, as smart glasses can capture the conversations, actions, and patient care process in videos, several participants expressed concerns about their personal liability and felt that they would be working under observation:

Maybe stuff you don't want recorded gets recorded and then a supervisor uses that against you. If they're trying to look for something, you know. It goes back to like the same things with police body cams, where, you know, stuff gets recorded without their knowledge. Sometimes it doesn't turn on when it's supposed to. So those would be the concerns I had and then who has access to see and hear what's recorded.

P8

## Discussion

### Principal Findings

In this study, we conducted semistructured interviews with prehospital care providers to understand the potential and affordances of smart glasses in EMS. We identified several potential application areas for smart glasses to support EMS work in the field, including (1) enhancing communication and consultation between distributed prehospital and hospital providers, (2) supporting patient data collection and documentation in a hands-free manner, (3) supporting decision-making and situation awareness, and (4) augmenting quality assurance and training. In the following section, situated in previous work, we discuss the feasibility of these potential applications and design considerations for realizing them. Major design considerations for the 4 identified application areas are summarized in Textbox 1.

**Summary of application areas and design considerations for applying smart glasses in emergency medical services.**

**Teleconsultation**

- Smart glasses should be designed to augment rather than replace current communication tools.
- Advanced mounting techniques are needed to make sure smart glasses sit steadily in front of the user’s eyes.

**Documentation**

- Novel techniques are needed to enable high performance of automatic speech recognition feature of smart glasses.
- More tests are needed to examine the usability and affordances of smart glasses in transcribing medical procedures.

**Decision support**

- Smart glass–based decision support interventions (eg, checklist) need to be designed such that they are dynamic and flexible enough to adapt to different patient scenarios.
- Artificial intelligence, computer vision, and smart glasses should be combined to automatically detect a patient’s signs and symptoms.

**Quality assurance and training**

- Patient data security and confidentiality must be maintained in accordance with Health Insurance Portability and Accountability Act regulations.
- Rules and policies need to be enacted to guide when video recording is allowed and who has the permission to watch the videos.

Communication and care coordination between prehospital and hospital teams are essential for safe, timely, and effective patient care. For example, the treatment of a pediatric patient with traumatic brain injury with a rapidly changing state of consciousness often requires a considerable level of knowledge and skills that EMS providers may not have. In this case, EMS providers may need to consult with a more experienced ED physician for advice (eg, what medications to administer or how to perform treatments that are critical to save the patient’s lives during ambulance transport) [45,46]. Furthermore, smooth communication can enable efficient joint decision-making between EMS and ED care providers with regard to the treatment plan, likely diagnoses, and appropriate destination of care [6,47-49]. Despite its critical role, this process remains ineffective [50-53]. This challenge is owing in part to the limitations of current communication mechanisms (eg, radio) because they limit multisensorial interaction—an important mechanism for ensuring smooth work and cooperation among collaborators [54]—between distributed care providers. Our study revealed that smart glasses were perceived to be a useful tool for EMS providers to connect with remote experts because they fulfill the need of visual supports through a *see-what-I-see* video. In fact, previous work has revealed the usefulness and feasibility of smart glasses in establishing remote expert support, such as in surgical telementoring [19-21], remote evaluation of patients experiencing acute stroke [22], and disaster telemedicine triage [23,24,55]. For example, a recent study [55] indicated that using smart glasses led to increased quality of triage during mass casualty incidents (MCIs). In addition, EMS providers reported satisfactory usability and good acceptance of the smart glass technology. However, there are a few considerations for deploying smart glasses in the out-of-hospital setting for use by EMS providers. For example, because smart glasses require a high-bandwidth cellular network for video calls, which is rare in some places (eg, rural areas and subway stations), smart glasses should only augment rather than replace current communication tools (eg, radio or cellular phone). However, with the rapid development of 5G technology and the proposition of building a dedicated broadband network for first responders (eg, FirstNet [56]), this limitation might be addressed in the near future. Another design consideration is regarding a common problem of using smart glasses for teleconsultation—difference in line of sight between distributed collaborators; that is, the remote expert could not always see what exactly the smart glass wearer’s local eyes were fixed on [57]. Therefore, more advanced mounting techniques are needed to ensure that smart glasses sit steadily in front of the user’s eyes even during excessive physical activities.

Collecting and documenting patient data in the field is a challenging and time-consuming task, which demands a significant portion of EMS professionals’ cognitive attention, thereby reducing their time spent on patient care [58]. Despite the increased adoption of EHR systems by EMS agencies, the real-time use of the EHR systems has faced many challenges. For example, as these systems are implemented on handheld devices such as tablets, EMS providers may not be able to use such devices in real time given the dynamic and hands-busy nature of prehospital care [10,11]. In addition, the use of handheld devices could increase the chance of cross-contamination [12]. Compared with handheld devices, smart glasses offer advantages such as hands-free operation, which has the potential to support real-time patient data collection and documentation. However, to date, very few studies have focused on supporting clinical documentation using smart glasses [28,59,60]. For example, a previous work [60] reported the design and evaluation of a smart glass app for chronic wound photography, which supported wound care nurses in documentation by enabling capture, tagging, and transfer of images to a patient’s EHR in a hands-free manner. In another study [59], researchers tested the patient’s acceptance and perception of their physician wearing smart glasses to connect with a remote scribe nurse who took notes during a clinical visit. These studies demonstrated the usefulness of smart glasses in supporting timely clinical documentation. However, they were conducted in settings where the working stress and noise level are considerably lower than the prehospital domain, which is often characterized as a noisy and messy environment that could affect the effective use of the voice recognition feature of smart glasses. In recent years, novel techniques have been developed to address this issue, such as a sensing and signal processing solution that enables high performance of automatic speech recognition of smart glasses [61]. To overcome challenges in realizing the documentation use for EMS, future research is needed to systematically test the usability and affordances of smart glasses in transcribing medical procedures while EMS providers perform them in noisy, dynamic, and fast-paced environments.

Similarly, very limited research has been conducted to examine the use of smart glasses for decision-making support. The most common feature reported in previous work is the presentation of a checklist or medical protocols on the glass screen. For example, in a recent study [62], researchers compared a smart glass–based checklist with conventional methods (ie, memory or poster) during surgical cases and found that smart glass–based checklists increased the completion rate to 100% and reduced the time required to execute the checklist and prepare the equipment. Another study [63] implemented triage algorithms on the smart glass platform to support the triage process during MCIs and reported that most EMS participants found the triage app to be useful or partially useful. Similarly, Follmann et al [55] found that smart glass can improve triage results during an MCI by showing the triage algorithms and by receiving support from a physician. These previous studies, despite not being extensive, illustrated that EMS teams could benefit greatly from smart glass checklists [3]. Given the unique characteristics of the EMS environment (eg, unpredictable clinical scenarios), smart glass–based checklists need to be designed such that they are dynamic and flexible enough to adapt to different patient scenarios, including less frequent but critical tasks [64,65]. In addition to the checklist application, future research can also explore combining artificial intelligence, computer vision, and smart glasses to automatically detect a patient’s signs and symptoms and recommend treatment options accordingly. For example, the artificial intelligence–powered smart glasses can help EMS providers to identify early signs of critical illnesses (eg, stroke) or hard-to-detect mechanisms of injury (eg, child abuse).

The use of smart glasses for quality assurance has received little attention so far, but it could become a new application area of smart glasses for not only EMS but also other medical domains. This use is realized mainly through the video recording feature. However, the challenging part is related to privacy issues. First, patient data security and confidentiality must be maintained in accordance with HIPAA regulations and other local, federal, or organizational policies. Second, patients and their surrounding environment (including bystanders) can be captured by smart glasses without their knowledge; that is, when transmitting videos from the field to the hospital for consultation. Therefore, EMS providers may need to obtain verbal or even written consent from the patient before using video recordings or calls. The smart glasses should also be designed to protect bystanders’ identities and privacy, such as automatically blurring their faces or recognizing their hand gestures for signaling consent (opt in) or disapproval (opt out) [66]. In addition, the glasses should clearly indicate when they are capturing videos to increase the awareness of bystanders; that is, through a light emitting diode strip [31,67]. Finally, as our participants explained, using smart glasses to capture EMS providers’ conversations, actions, and patient care processes in videos could easily trigger their privacy and liability concerns. Given these privacy issues and considerations that are entailed in using smart glasses to record videos, organizational and national rules and regulations should be in place to provide guidance as to when video recording is allowed and who has the permission to watch the videos.

Regardless of the application areas, users should be able to interact with the smart glasses in an intuitive manner without disrupting their work practice. Our data shows that voice commands and hand gestures are preferred over touch pad because of their *hands-free* advantage. Despite the benefits, noisy environment can pose challenges in using voice commands, whereas fast-moving ambulances can affect the use of hand gestures. Therefore, as our participants stated, it is critical for them to have the option to use a mix of interaction mechanisms at any time while using the smart glass app.

Our study also revealed some other considerations that need full attention before deploying the smart glass technology in EMS. For example, as previous work has pointed out [35], it is important to ensure that the device’s battery can last long enough for care providers to use it throughout a shift. In addition, interoperability was cited as a critical consideration for the successful implementation of smart glasses in the field. That is, the smart glass device should be integrated with existing systems (eg, vital signs monitor and EHR system) to allow seamless data exchange. Finally, the medicolegal aspects of smart glasses could impact the real use of this novel technology. For example, when using smart glasses as a telemedicine tool, the medicolegal obligations are placed on both distant and local emergency care providers. In addition, the patient must be informed about the nature, purpose, and use of the smart glass device and what benefits this technology can offer to them [68]. Any potential breach in patient’s privacy and confidentiality must be addressed to enhance patient-centered care.

### Limitations

This study has several limitations that need to be noted. First, the interviews were not conducted in person. Although we gave video and live demonstrations of the smart glass device, participants did not get an opportunity to use it. This limitation could have impacted their views on this technology. Second, participants were recruited from hospital-based EMS agencies in an urban area of the east coast region in the United States. Therefore, the user perceptions were based on how they operate locally, which may be different from other places (eg, rural areas and other regions of United States) and other types of EMS providers (eg, fire department–based or volunteer-based), let alone other countries. Therefore, the results may not be generalizable to all types of EMS agencies worldwide. More work in other regions and countries is needed to supplement the findings of this study. Third, user opinions and needs were collected only through interviews. We neither asked the participants to use the device nor tested the effectiveness and usefulness of this technology under different case scenarios. This may have impacted the participants’ views on smart glass. Additional studies, such as participatory design workshop and usability evaluation, will be conducted in the future to elicit additional design insights about smart glasses for EMS. In addition, it is critical to conduct simulated scenarios to test the efficiency and effectiveness of smart glasses for different application areas. Finally, our participants were mostly male. Female participants may have different opinions and preferences. In our future work, we will include as many female EMS providers as possible.

### Conclusions

In this study, we conducted semistructured interviews with EMS providers to learn their opinions, needs, and concerns regarding the use of smart glasses in their daily work. Our results identified four potential application areas in which smart glasses can play an essential role, including enhancing teleconsultation between distributed prehospital and hospital providers; semiautomating patient data collection and documentation in real time; aiding decision-making and situation awareness; and finally, augmenting quality assurance and training. We also found that voice commands and hand gestures were preferred over the built-in touch pad for system navigation. Although EMS providers consider smart glasses as a suitable technological means for prehospital work, several issues and user concerns, such as hardware limitations, human factors, reliability, workflow, interoperability, and privacy, need to be thoroughly addressed to ensure its successful uptake and implementation. Finally, we identified several design considerations for realizing the applications of smart glasses in EMS.

## Appendix

Multimedia Appendix 1 Interview guide.

## Abbreviations

ED: emergency department
EHR: electronic health record
EMS: emergency medical services
HIPAA: Health Insurance Portability and Accountability Act
MCI: mass casualty incident
