# Supplementary material for: Applications and User Perceptions of Smart Glasses in Emergency Medical Services: Semistructured Interview Study
Source: JMIR Hum Factors. 2022 Feb 28;9(1):e30883. doi: 10.2196/30883 (PMC8922155; doi:10.2196/30883)
Supplement: Multimedia Appendix 1 [file humanfactors_v9i1e30883_app1.docx]

**Interview Guide**

**Background/Experience**

What is your title OR official role on the EMS team?

How many years of experience in this job/role?

**Workflow**

Please describe a typical day.

Please describe the typical EMS workflow.

**Challenges**

What kinds of challenges or problems can you think of in your work?

- If mentioned challenges related to documentation, probe the following questions:
  - Are you satisfied with the current documentation system?
  - What barriers can you think of in terms of using the documentation system in real-time?
  - What is your workaround?
  - What information do you usually collect?
- If mentioned challenges related to dispatching, probe the following questions:
  - Is the information provided by the dispatcher sufficient for you to anticipate patient needs?
  - Can you think of any problem that might exist in the communication process with the dispatcher?
- If mentioned challenges related to communicating with remote physicians, probe the following questions:
  - What tools are you currently using to communicate with the receiving hospital?
  - How challenge is it to precisely describe patient situation using current communication tools?
  - What information do you usually share with the receiving hospital?

**Perceptions of Using Smart Glasses**

[Demonstrate smart glasses and three interaction modalities (voice command, touch pad, and hand gesturing)]

What aspects of your work can be facilitated/supported by smart glasses? How helpful is it to your work? And why?

Which interaction method would you prefer to use when interacting with the smart glass?

What concerns do you have regarding the use of smart glasses in your daily work?

What concerns do you think patients might have if you use the smart glasses?

Do you think smart glasses could be easy to use and integrate into your current work practice?

Do you think smart glasses can impede/interfere your work? If so, how?

Are you willing to use this technology?
